# Effects of family-based dignity intervention and expressive writing on anticipatory grief in family caregivers of patients with cancer: a randomized controlled trial

**DOI:** 10.1186/s12888-023-04715-x

**Published:** 2023-04-01

**Authors:** Tahereh Najafi Ghezeljeh, Naima Seyedfatemi, Jafar Bolhari, Naser Kamyari, Masoud Rezaei

**Affiliations:** 1 Nursing and Midwifery Care Research Center, School of Nursing and Midwifery, Iran University of Medical Sciences, Tehran, Iran; 2 Spiritual Health Research Center, School of Behavioral Sciences and Mental Health, University of Medical Sciences, Tehran, Iran; 3 Department of Public Health, School of Health, Abadan University of Medical Sciences, Abadan, Iran

**Keywords:** Anticipatory grief, Caregivers, Cancer, Dignity, Expressive writing

## Abstract

Family caregivers of dying cancer patients may suffer from grief experiences and bereavement complications. Previous studies have proposed some psycho-emotional interventions for the management of these complications. However, little attention has been given to family-based dignity intervention and expressive writing. This study was conducted to examine the effects of family-based dignity intervention and expressive writing, combined and alone, on anticipatory grief in family caregivers of dying cancer patients. This was a randomized controlled trial, in which 200 family caregivers of dying cancer patients were randomly assigned to four intervention groups: family-based dignity intervention (*n* = 50), expressive writing intervention (*n* = 50), combined family-based single dignity intervention and expressive writing (*n* = 50), and control group (*n* = 50). In three times (baseline, 1 week, and 2 weeks after the interventions), anticipatory grief was assessed by a 13-item anticipatory grief scale (AGS). Finally, we found a significant reducing effect of family-based dignity intervention on AGS (-8.12 ± 1.53 vs. -1.57 ± 1.52, *P* = 0.01) and its subscales including behavioral (-5.92 ± 0.97 vs. -2.17 ± 0.96, *P* = 0.04) and emotional (-2.38 ± 0.78 vs. 0.68 ± 0.77, *P* = 0.03) subscales compared to the control group. However, no significant effect was seen for expressive writing intervention and combined interventions of expressive writing and family-based dignity intervention. In conclusion, family-based dignity intervention may be a safe intervention for relieving anticipatory grief among family caregivers of dying cancer patients. Additional clinical trials are needed to confirm our findings. Registration number: IRCT20210111050010N1. Trial registration date:2021–02-06.

## Introduction

Cancer is a chronic disease and a major health problem that is among the most feared life-threatening diseases despite considerable advances in its treatment [1, 2]. This disease imposes a high burden on patients and their families [3]. As cancer progresses, patients became physically and psychologically weaker, and their dependency on family increases [4]. Family caregivers of cancer patients may be involved in not only the patients’ sufferings but also hospital policies, economic difficulties, and social challenges [5–7]. Therefore, family caregivers, particularly those who take care of dying cancer patients, may be at risk of psychological disorders and feelings of helplessness, hopelessness, and anticipatory grief [8]. There is evidence indicating that 35% of family caregivers are affected by psychological disorders [9]. Therefore, finding appropriate strategies to improve the mental health of these people is of great importance.

The religious culture of Iranian caregivers makes a strong relationship between caregivers and patients [10]. Therefore, they willingly accept all patient's problems and look at their responsibilities as moral commitment and divine duty [11]. Nevertheless, this religious belief may cause them to hide their needs and caregiving problems [12]. Moreover, there are no specific social organizations in Iran to support caregivers and diminish their problems [13]. Therefore, family caregivers in Iran, compared with those from developed countries, may be at a higher risk of psychological disorders or anticipatory grief.

Anticipatory grief is a usual psychological problem among family caregivers of cancer patients [14]. This may occur when caregiver think about the threat of death or separation, while the cancer patient is physically present and needs care [15]. It seems that anticipatory grief is a safeguard against the impact of a sudden death that helps caregivers to tolerate separation grief [16]. However, it must be kept in mind that anticipatory grief is a stressful condition that may be associated with an increased risk of psychological distress among caregivers [17]. Furthermore, anticipatory grief in caregivers may affect cancer patients and their treatment process as well as caregiving quality [18]. Therefore, management of this feeling has a beneficial effect on the health status of caregivers and cancer patients [19].

Some psychological interventions have been proposed to help critically ill patients and their caregivers to cope with their difficulties [19–22]. Among them, family-based dignity intervention and expressive writing received great attention [20, 23]. Family-based dignity intervention is a spiritual psychological intervention taken from dignity therapy methods [23]. This supportive intervention helps caregivers to strengthen their sense of hope in themselves and gives them an opportunity to talk about their successes, aspirations, and plans [23]. Expressive writing intervention includes sessions of solitary and unlimited writing about positive and negative feelings and experiences caused by stressful events [24, 25]. Overall, talking about successes, aspirations, and plans and expressing feelings through writing is an appropriate strategy to enhance well-being and may promote the ability of caregivers to cope with chronic grief [25].

Previous studies have mainly focused on the effect of family-based dignity intervention on the mental health of dying cancer patients [26, 27] and less attention has been paid to caregivers of these patients. In a randomized controlled trial (RCT), Xiao et al. reported that family-oriented dignity therapy relieved existential distress, depressive symptoms, and spiritual well-being among patients with lung cancer [26]. The beneficial effect of dignity therapy on dying cancer patients was also reported in another study [27]. Based on our literature review, we found no study investigating the effect of family-based dignity intervention on caregivers of dying cancer patients. In terms of expressive writing, we found only one study in which Leung et al. concluded that this intervention was a safe and cost-effective supportive intervention for caregivers of cancer patients [20]. However, anticipatory grief of caregivers was not assessed in that study. In addition, we are aware of no study that examined the combined effects of family-based dignity intervention and expressive writing on caregivers of cancer patients. Therefore, the current study was conducted to assess the effects of family-based dignity intervention and expressive writing, combined and alone, on anticipatory grief of caregivers of dying cancer patients.

## Methods

### Participants

This randomized clinical trial was conducted in Tehran, Iran, during March 2021 to April 2022. Details on participants, study design, and methods used to assess outcome variables were published previously [28]. In the current study, we recruited family caregivers of dying cancer patients who were referred to the oncology center of Firoozgar hospital affiliated to the Iran University of Medical Sciences, Tehran, Iran, to receive medical and palliative care for their cancer patients.

### Inclusion criteria

We included caregivers with the following criteria: 1) caregivers who were the first-degree relatives of cancer patients and had the most responsibility for caregiving of them during the last 3 months, 2) those who were caregiving of cancer patients who were dying or critically ill according to the opinion of the treating physician, and 3) caregivers aged ≥ 18 years.

### Non-inclusion and exclusion criteria

We did not include caregivers who have a history of psychological disorders and those who had a history of death among their first-degree relatives. Also, caregivers who were not first-degree relatives of dying cancer patients and those who had experienced psychological interventions (particularly dignity and expressive writing interventions) during the last 6 months were not included.

### Exclusion criteria

We excluded caregivers who were not willing to continue each phase of this study. Those caregivers whose cancer patients died during the study were excluded as well.

### Ethics

We explained the aims and implementation process of the current study to all caregivers and they could optionally accept to participate in this study. The ethics committee of the Iran University of Medical Sciences approved the study (code: IR.IUMS.REC.1399.1097). Moreover, this study was registered in the Iranian Registry of Clinical Trials (www.irct.ir) with code IRCT20210111050010N1.

### Sample size calculation

We used the following formula to calculate the required sample size [29]. This calculation was done based on the type I error of 5% (α = 0.05), type II error of 20% (β = 0.20, power = 80%), and anticipatory grief score as the key variable. The mean and SD of anticipatory grief scores were obtained from the study of Fowler et al. [30].

$$n=\frac{{2[(\mathrm{a}+\mathrm{b})}^{2}\times {\upsigma }^{2}]}{{({\mu }_{1}-{\mu }_{2})}^{2}}$$

*n* = sample size in each group

μ_1_ = mean for anticipatory grief score in group 1 (considered as 78.17 based on the study of Fowler et al. [30])

μ_2_ = mean for anticipatory grief score in group 2 (considered as 64.49 based on the study of Fowler et al. [30])

σ = variance (SD) for the mean of anticipatory grief score which was considered 23.1 (the average SDs reported for anticipatory grief score in the 2 groups of Fowler et al. study [30]).

a = conventional multiplier for alpha = 0.05 that was 1.96

b = conventional multiplier for power = 0.80 that was 0.842

Overall, based on the formula and given a 10% drop-out in each group, we needed a sample size of 50 caregivers for each intervention group.

### Study design

After selecting 200 family caregivers based on the inclusion criteria, they were randomly assigned to one of the four intervention groups: family-based dignity intervention (group 1, *n* = 50), expressive writing intervention (group 2, *n* = 50), combined family-based dignity intervention and expressive writing (group 3, *n* = 50), and controls (group 4, *n* = 50). Allocation concealment was performed using the block randomization method. Complete information on block randomization is presented in our study protocol published previously [28]. In brief, we used six blocks, each with a block size of 4 (A: group 1, B: group 2, C: group 3, and D: group 4), and the order of the letters in these blocks was different (e.g. ABCD, ACDB, and etc.). Then, a code ranging between 1 and 6 was assigned to each of these six blocks. For allocating each four caregivers, at first, we randomly selected one of the six blocks using a six-sided dice, and then, caregivers were assigned to the four intervention groups according to the order of letters in the selected block. Until all groups became complete, the random allocation continued. Random allocation was done by a person who was unaware of the aim of our study. After the random allocation, an appropriate time was set for all caregivers to participate in a session related to the interventions (family-based dignity intervention and expressive writing). Before the session and 1 and 2 weeks after the session, anticipatory grief was evaluated using the 13-item anticipatory grief scale.

### Family-based dignity intervention

Family-based dignity intervention (FBDI) was performed for each caregiver in a 60–90-min interview session by a trained nurse who was experienced in counseling. This intervention was conducted based on the protocol designed by Ho and his colleagues [31]. The interview session took place in a private room in the oncology center of Firoozgar hospital, Tehran, Iran. In this session, caregivers were asked to answer 12 open-ended questions to get their reflections. Details on the questions are presented in Table 1. The questions focused on eliciting caregivers’ experiences of living with a cancer patient before and after the diagnosis of cancer. Also, some questions were aimed to help caregivers to review the beautiful memories of living with a cancer patient and express their hopes, wishes, and desired expectations. After asking each question, the nurse recorded the caregivers’ responses to the patient’s life experiences in the family context. In addition, the nurse helped caregivers structure and organize their thoughts, connect sequences of events, facilitate disclosure of cherished memories, and encourage the expression of appreciation and reconciliation. FBDI was quickly transcribed verbatim and shaped into a coherent narrative by the nurse using a formatted editing process. Finally, the nurse and caregiver reviewed the transcript to ensure it conveys the caregiver’s overall message.

**Table 1:** Question framework of Family Dignity Intervention

1. Tell me a little about your life history with your loved one; what are some of the most important and memorable times you had together? When did you feel most alive with your loved one?
2. How has your relationship with your loved one influenced your life?
3. What are some things you want your loved one to know about you, or to remember about you?
4. What do you think are your loved one’s most important and meaningful accomplishments in life (family, career, community)?
5. What do you appreciate most about your loved one?
6. What do you think your loved one is most proud of you for, or appreciates about you?
7. Are there particular things that you want to thank your loved one for?
8. Are there particular things that you like to ask forgiveness for, or offer forgiveness for?
9. What teachings, advice, or words of guidance have you received from your loved one, and would like to pass on to other family members?
10. What are your hopes and dreams for future for your loved one, yourself and your family?
11. In creating this permanent record, are there other things that you would like to include?
12. Before the session ends, are there things that you would like to take time to say again?

### Expressive writing intervention

We used the Pennebaker method to perform expressive writing intervention [24]. In a previously set session, caregivers were instructed by a trained nurse in order to do the writing. Caregivers were instructed to ‘‘really let go and explore their very deepest emotions and thoughts”. We told caregivers to write about their negative and positive family memories and describe their experiences of caregiving in the present and past time. Each caregiver was asked to consider four areas for expressive writing: emotional disclosure, cognitive appraisal, benefit finding, and looking to the future. Caregivers were asked to do writing three times (lasting 20 min) in a week. During that week, we reminded the writing process using a phone call. They were assured that they do not need to worry about sentence structure or grammatical errors when writing. After one week of opportunity for expressive writing, the manuscript of caregivers was received.

### Combined family-based dignity intervention and expressive writing

For the combined intervention, at first, we took place the session related to the family-based dignity intervention, and then, training on expressive writing was delivered to caregivers. One week after the last session, the manuscript of caregivers was received.

### Control group

Caregivers in the control group, as well as those in the intervention groups, received routine care such as family counseling and meaning therapy. This was a standard protocol for all caregivers in the center we performed the current study. This protocol was done by a palliative medicine specialist. In brief, meaning therapy is an integrative and positive existential approach for counseling and psychotherapy [32].

### Anticipatory grief scale

In the current study, we used a short form of the anticipatory grief scale (AGS) to assess the anticipatory grief of caregivers before and after the interventions. This scale was introduced by Holm et al. [33] in 2019 and consisted of 13 items measuring anticipatory grief on a Likert scale, ranging from 1 (strongly disagree) to 5 (strongly agree). By summing up the items, a total score ranging between 13 and 65 was obtained. Higher scores indicate higher anticipatory grief in caregivers of cancer patients. This scale was specifically designed for family caregivers of cancer patients. In addition, it consists of two subscales, named “*Behavioral reactions”* (items 1 to 8) and “*Emotional reactions”* (items 9 to 13), which capture the behavioral and emotional reactions of grief in caregivers participating in palliative care.

## Translation, validity, and reliability of AGS-13

### Translation

Since the AGS-13 was not used in Iran, we translated it to Persian based on the method proposed by Guillemin [34]. At first, we got permission from its developer to use and translate the AGS-13 (Holm et al.) [33]. Then, the English version of the instrument was translated into Persian by two independent health professionals who were fluent in English and Persian languages and familiar with the concepts of the questionnaire. After that, an expert panel assessed the translations and selected the best translation of each item. Then, the Persian form of this scale was translated back to English by two other qualified persons and the best backward translation was chosen by the same expert panel afterward. The final backward translation form was sent to the developer (Dr. Holm) and he confirmed the adequacy and transparency of words and concepts. Therefore, the confirmed Persian version of AGS-13 was used in the current study.

### Validity

Content validity (CV) was applied to assess the validity of AGS-13. The assessment of the CV was done using two qualitative and quantitative methods. In the qualitative method, we sent the Persian version of the AGS-13 to 11 experts in the fields of instrumentation and psychometric measurement, community health nursing, and specialists in the field of psychology and oncology and received their opinions in terms of grammar, wording, item allocation, and scaling. In the quantitative method, CV was examined using Content Validity Ratio (CVR) and Content Validity Index (CVI) [35, 36]. According to Waltz et al. study, CVR and CVI of > 0.79 indicate appropriate validity of a questionnaire. In the current study, the obtained CVR and CVI were ≥ 0.80 and ≥ 0.90, respectively, for all items of the AGS-13 indicating the appropriate validity of AGS-13.

### Internal consistency

We calculated Cronbach’s alpha coefficient using SPSS software (version 18) to assess the internal consistency. This coefficient for total AGS and behavioral and emotional subscales was 0.89, 0.81, and 0.89, respectively, which indicated the appropriate internal consistency of AGS-13.

### Test–retest reliability

To measure the stability of the AGS-13, 30 caregivers were asked to fill out the scale two times with a two-week interval. These caregivers did not participate in the main study. The intra-class correlation coefficients (ICC) for total AGS as well as its subscales were > 0.98 indicating the appropriate reliability of AGS-13.

### Baseline assessment

At baseline, we collected information on age, gender, marital status, education, occupation, economic status, place of residence, disease history, and time spent on caregiving patients. In addition, for cancer patients who had been cared for by caregivers, data on age, gender, marital status, occupation, health insurance, economic status, duration of cancer, treatment methods, and cancer type were collected using a research-made questionnaire. Data on caregivers and cancer patients were obtained through face-to-face interviews with caregivers.

### Statistical analysis

The Kolmogorov–Smirnov test was used to examine the normal distribution of quantitative variables [37]. According to the test, AGS scores had a normal distribution. The analyses were performed on the basis of an intention-to-treat (ITT) approach. Therefore, missing values were treated according to the last-observation-carried-forward method. To detect differences in quantitative and categorical variables across the 4 intervention groups, we used the analysis of variance (ANOVA) and Chi-square test, respectively. To determine the effect of family-based dignity intervention and expressive writing on anticipatory grief, we used repeated-measures ANOVA. In this analysis, the 4 intervention groups were considered as the between-subjects factor and the time points (Before and 1 and 2 weeks after the interventions) were considered as the within-subjects factor. Also, to assess differences between the 4 intervention groups in terms of mean changes in anticipatory grief, the multivariate analysis of covariance (ANCOVA) was used by considering baseline measurements as covariates. All statistical analyses were conducted using the SPSS software version 18 (SPSS, Inc. Chicago, IL, USA). *P*-value < 0.05 was considered a significant level.

## Results

**Fig. 1:**
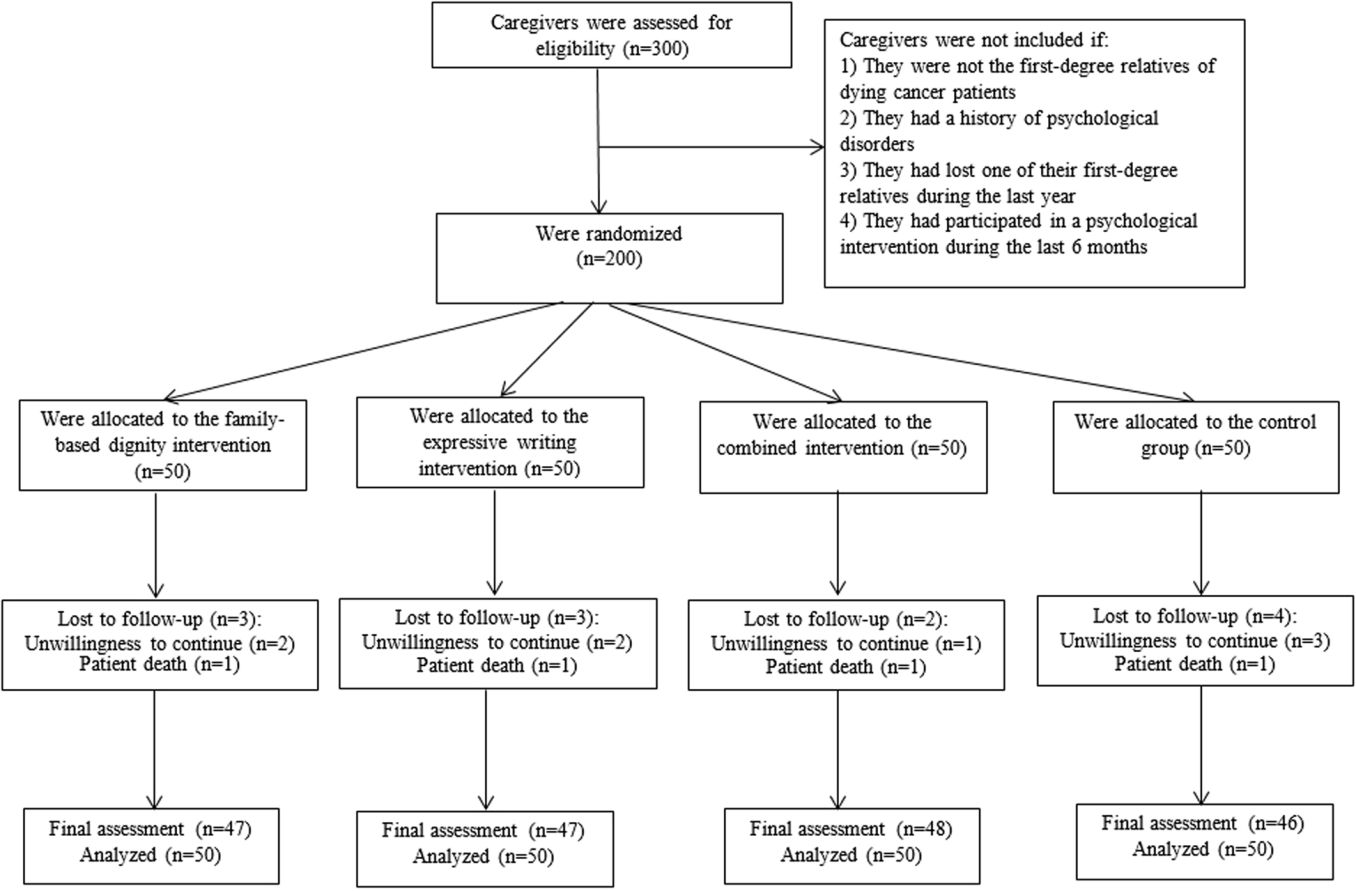
Flowchart of study

Of the 200 caregivers included at baseline, two participants in the family-based dignity intervention group, two participants in the expressive writing group, one participant in the combined intervention group, and three participants in the control group were excluded because they were not willing to continue the study. In addition, four caregivers (one in each group) were excluded because their patients died during the follow-up. Finally, a total of 188 caregivers (47 in the family-based dignity intervention group, 47 in the expressive writing group, 48 in the combined intervention group, and 46 in the control group) completed the study. However, the statistical analyses were performed on all 200 caregivers on the basis of an intention-to-treat approach. In this approach, missing values for the 12 excluded caregivers were determined on the basis of the last-observation-carried-forward method. Figure 1 shows the study flowchart.

Baseline characteristics of caregivers across the four intervention groups are presented in Table 2. The distribution of gender was significantly different across the four intervention groups; such that caregivers in the combined intervention group were more likely to be female compared with other groups. No other significant differences were found in terms of demographic variables, disease history, and time spent for caring the cancer patient across the four intervention groups. General characteristics of cancer patients who were cared for by caregivers are shown in Table 3. We found no significant differences between the four intervention groups in terms of demographic characteristics, cancer type, treatment methods, and cancer stage of patients who were cared for by caregivers.

**Table 2:** Baseline characteristics of caregivers across the 4 intervention groups

Variables	FDI group	EWI group	FDI + EWI group	Control group	*P*-value^*^
Age (year)	38.96 ± 11.45	37.78 ± 9.61	40.24 ± 11.93	38.30 ± 10.26	0.69
≥ 35 years (%)	66.0	56.0	70.0	56.0	0.35
Female (%)	52.0	62.0	80.0	58.0	0.02
Married (%)	72.0	72.0	76.0	80.0	0.75
Relative to patient					0.17
Children	58.0	48.0	44.0	56.0	
Brother/sister	16.0	20.0	8.0	20.0	
Parents	18.0	14.0	20.0	14.0	
Other	8.0	18.0	28.0	10.0	
University educated (%)	44.0	36.0	26.0	30.0	0.25
Occupation (jobless) (%)	56.0	70.0	70.0	64.0	0.40
Economic status (weak) (%)	14.0	24.0	22.0	28.0	0.71
Urban (%)	82.0	86.0	86.0	82.0	0.89
Diabetes/HTN (%)	10.0	12.0	22.0	18.0	0.32
Living with patient (%)	62.0	68.0	72.0	58.0	0.44
Time spending for patient (> 8 h)	96.0	96.0	96.0	88.0	0.50

Data are presented as mean (± SD) or percent

Abbreviations: *FDI* Family-based dignity intervention, *EWI* Expressive writing intervention, *HTN* Hypertension

^*^Obtained from the one-way analysis of variance (ANOVA) or Chi-square test, where appropriate

**Table 3:** Baseline characteristics of cancer patients cared for by caregivers across the 4 intervention groups

Variables	FDI group	EWI group	FDI + EWI group	Control group	*P*-value^*^
Age (year)	46.68 ± 18.73	46.66 ± 19.53	48.12 ± 17.86	47.66 ± 18.09	0.97
≥ 35 years (%)	70.0	68.0	80.0	74.0	0.54
Female (%)	58.0	48.0	50.0	56.0	0.64
Married (%)	62.0	74.0	68.0	70.0	0.62
Having health insurance (%)	94.0	94.0	94.0	92.0	0.97
Occupation (jobless) (%)	34.0	36.0	46.0	30.0	0.39
Economic status (weak) (%)	22.0	38.0	40.0	42.0	0.40
Cancer diagnosis time (< 6 months)	12.0	18.0	12.0	26.0	0.69
Cancer therapy					0.79
Chemotherapy (%)	54.0	52.0	52.0	58.0	
Radiotherapy (%)	6.0	8.0	12.0	14.0	
Operation history (%)	58.0	58.0	62.0	52.0	0.79
Disease stage (stage 4) (%)	38.0	46.0	46.0	42.0	0.80
Cancer type (%)					0.43
Lung	4.0	8.0	6.0	10.0	
Colorectal	16.0	12.0	22.0	14.0	
Breast	16.0	8.0	6.0	4.0	
Leukemia	8.0	6.0	10.0	12.0	
Gastric	6.0	8.0	4.0	10.0	
Liver	8.0	6.0	4.0	4.0	
Pancreases	2.0	4.0	10.0	10.0	
Ovarian	4.0	6.0	4.0	4.0	
Prostate	2.0	6.0	6.0	2.0	
Sarcoma	18.0	22.0	14.0	14.0	
Esophageal	8.0	6.0	4.0	4.0	
Brain	4.0	2.0	0	2.0	
Bladder	2.0	0	2.0	10.0	
Lymphoma	2.0	4.0	8.0	0	

Data are presented as mean (± SD) or percent

Abbreviations: *FDI* Family-based dignity intervention, *EWI* Expressive writing intervention

^*^Obtained from the one-way analysis of variance (ANOVA) or Chi-square test, where appropriate

Total scores of AGS and its subscales at baseline, 1 and 2 weeks after the interventions in family caregivers of dying cancer patients are shown in Table 4. Compared with the baseline, the total scores of AGS and behavioral subscale significantly reduced in weeks 1 and 2 in all intervention groups (P-time < 0.001); however, these reductions in week 2 were lower than in week 1. Comparing the reductions across the 4 intervention groups, we fail to find any significant difference in the scores of AGS (P-group = 0.14) and behavioral subscale (P-group = 0.21). Also, we found no significant interaction between time and group about changes in the total scores of AGS (P-time*group = 0.20) and behavioral subscale (P-time*group = 0.19). In terms of emotional subscale score, we found no significant findings in the within- and between-group comparisons (Ptime = 0.09, P-group = 0.11) as well as in the interaction between time and group (P-time*group = 0.56).

**Table 4:** Mean AGS at baseline, one week and two weeks after the interventions among family caregivers of dying cancer patients

	FDI group	EWI group	FDI + EWI group	Control group	*P*-value^*^		
					Time	Group	Time*group
Total AGS					< 0.001	0.14	0.20
Baseline	45.40 ± 13.19	48.54 ± 14.06	49.28 ± 12.15	48.80 ± 12.78			
Week 1	41.86 ± 9.93	43.18 ± 12.32	42.94 ± 8.23	44.50 ± 14.71			
Week 2	38.74 ± 10.39	44.00 ± 12.39	43.74 ± 10.08	46.78 ± 15.09			
Behavioral subscale					< 0.001	0.21	0.19
Baseline	29.02 ± 8.13	30.84 ± 8.44	31.38 ± 7.96	30.84 ± 8.27			
Week 1	25.92 ± 6.70	27.28 ± 7.40	26.12 ± 5.87	27.04 ± 8.88			
Week 2	23.90 ± 7.44	27.46 ± 7.48	27.12 ± 6.60	28.50 ± 9.44			
Emotional subscale					0.09	0.11	0.56
Baseline	16.38 ± 6.58	17.70 ± 6.23	17.90 ± 5.62	17.96 ± 5.66			
Week 1	15.94 ± 4.85	15.90 ± 6.25	16.82 ± 4.50	17.46 ± 6.49			
Week 2	14.84 ± 4.50	16.54 ± 6.18	16.62 ± 4.89	18.28 ± 6.68			

Data are presented as mean ± SD

Abbreviations: *FDI* Family-based dignity intervention, *EWI* Expressive writing intervention, *AGS* Anticipatory grief scale

^*^Obtained from the repeated measures ANOVA

Table 5 shows adjusted mean changes in the scores of AGS and its subscales in caregivers across the four intervention groups. In the analysis, baseline scores of AGS and its subscales were adjusted. Considering the changes in AGS and its subscales between the baseline and week 2, we found significant differences across the 4 intervention groups. The two-by-two comparison showed a significant reduction in the total scores of AGS (-8.12 ± 1.53 vs. -1.57 ± 1.52, *P* = 0.01), behavioral (-5.92 ± 0.97 vs. -2.17 ± 0.96, *P* = 0.04), and emotional (-2.38 ± 0.78 vs. 0.68 ± 0.77, *P* = 0.03) subscales in the family-based dignity group compared with the control group. When comparing the mean changes between the baseline and week 1 across the four intervention groups, no significant differences were seen. However, considering the mean changes between weeks 1 and 2, we found a significant effect of family-based dignity intervention, compared with the control group, on the total scores of AGS (-3.30 ± 1.25 vs. 2.33 ± 1.24, *P* = 0.01) and behavioral subscale (-2.06 ± 0.93 vs. 1.47 ± 0.93, *P* = 0.04). This effect was not significant for the emotional subscale. Regarding expressive writing and combined intervention, we found no significant effect on AGS and its subscales when comparing the times with each other.

**Table 5:** Adjusted mean changes of AGS among family caregivers of dying cancer patients in the four intervention groups

	FDI group	EWI group	FDI + EWI group	Control group	*P*-value^*^
Total AGS					
Week 2-baseline	-8.12 ± 1.53^a^	-4.24 ± 1.52	-4.82 ± 1.52	-1.57 ± 1.52	0.02
Week 1-basline	-4.81 ± 1.35	-5.10 ± 1.34	-5.72 ± 1.34	-3.91 ± 1.34	0.81
Week 2-week 1	-3.30 ± 1.25^a^	0.86 ± 1.25	0.89 ± 1.25	2.33 ± 1.24	0.01
Behavioral subscale					
Week 2-baseline	-5.92 ± 0.97^a^	-3.21 ± 0.96	-3.80 ± 0.97	-2.17 ± 0.96	0.049
Week 1-basline	-3.86 ± 0.86	-3.40 ± 0.86	-4.82 ± 0.86	-3.64 ± 0.86	0.66
Week 2-week 1	-2.06 ± 0.93^a^	0.19 ± 0.93	1.03 ± 0.93	1.47 ± 0.93	0.04
Emotional subscale					
Week 2-baseline	-2.38 ± 0.78^a^	-1.00 ± 0.77	-0.96 ± 0.77	0.68 ± 0.77	0.053
Week 1-basline	-1.09 ± 0.72	-1.67 ± 0.71	-0.83 ± 0.71	-0.22 ± 0.71	0.54
Week 2-week 1	-1.28 ± 0.74	0.67 ± 0.74	-0.13 ± 0.74	0.90 ± 0.74	0.15

Data are presented as mean ± SE adjusted for baseline values of AGS

Abbreviations: *FDI* Family-based dignity intervention, *EWI* Expressive writing intervention, *AGS* Anticipatory grief scale

^*^Obtained from the one-way analysis of covariance (ANCOVA)

^a^ Significant compared with the control group

## Discussion

In the current study, we found a significant reducing effect of family-based dignity intervention on AGS and its subscales compared with the control group. On the other hand, family-based dignity intervention could reduce anticipatory grief among caregivers of dying cancer patients. However, we found no significant effect for expressive writing intervention and combined interventions of expressive writing and family-based dignity intervention. To the best of our knowledge, this is the first study that examined the combined effects of family-based dignity intervention and expressive writing on caregivers of dying cancer patients.

Since family caregivers of dying cancer patients have inadequate social support and face several problems related to their patients, they may suffer from different psychological disorders such as depression, anxiety, and psychological distress [14, 38]. Anticipatory grief is one of the most important complications of individuals that care for dying patients [39]. Thinking about separation and loneliness may initiate a grief reaction in caregivers when their patient is physically present and needs a lively caregiver [15]. Therefore, the management of anticipatory grief can increase the quality of caregiving and also improve caregivers’ health [40].

Recently, it has been shown that some psychological interventions such as family-based dignity intervention and expressive writing have a beneficial effect on psychological disorders in dying patients [26, 27]. However, no study examined the effects of these interventions on anticipatory grief in caregivers of dying cancer patients. In the current study, we found that family-based dignity intervention had a beneficial effect on anticipatory grief among caregivers of dying cancer patients. In a clinical trial, Wang et al. reported that family participatory dignity therapy improved anxiety and depression and enhanced family cohesion and adaptability among caregivers of patients with hematologic malignancies [41]. In addition, family participatory dignity therapy on cancer patients had a positive effect on promoting patients' hope and spiritual well-being [41]. In another clinical trial, family-oriented dignity intervention had a potential role to relieve existential distress and depressive symptoms and improve spiritual well-being in patients with lung cancer [26].

In total, our study and results from the previous studies confirmed that caregivers can benefit from family-based dignity intervention. This positive effect might be explained by that the family-based dignity intervention gives caregivers an opportunity to release pressure and express their true feelings and concerns. This approach may have a relieving effect on anxiety and depression associated with caregiving work [41]. Moreover, family-based dignity intervention may buffer emotional distress among patients and their caregivers by the reminiscent of old memories and increment mutual understanding and support [42].

In the current study, expressive writing intervention had no significant effect on anticipatory grief and its subscales in caregivers of dying cancer patients. However, previous studies reported beneficial effects of expressive writing on caregivers of cancer patients. In a clinical trial, Leung et al. reported that expressive writing could enhance the ability of caregivers to overcome their difficulties in caregiving of cancer patients [20]. In another clinical trial, written emotional disclosure could reduce caregivers' depression compared with the control group [43]. The observed disparity between our study and previous studies might be due to the different outcome variables evaluated following family-based dignity intervention. In none of the previous studies, anticipatory grief was assessed. Furthermore, different types of expressive writing interventions are another reason for the disparity. For instance, in the Leung et al. study that reported a beneficial effect of expressive writing, caregivers did expressive writing in the context of a group of 4 to 8 caregivers, while in the current study, caregivers did that alone. Moreover, in the Leung et al. study, caregivers could share their difficulties. Therefore, it seems that doing expressive writing in a group is more effective than doing alone.

The lack of significant effect of the combined intervention (family-based dignity + expressive writing) on anticipatory grief might be justified by the lack of significant effect of expressive writing. In our study, for caregivers in the combined intervention, we first hold the family-based dignity sessions, and then, caregivers were asked to do writing within a week after the dignity intervention. Therefore, the beneficial effect of family-based dignity intervention may have been attenuated by the expressive writing process. Further studies are needed to confirm our findings on the effect of expressive writing on anticipatory grief among cancer caregivers.

It seems that anticipatory grief might be more affected by those interventions that participants can communicate with consultants or other participants. In the current study, in the family-based dignity intervention, the communication between interviewers (nurses) and caregivers in an interview session might help caregivers to sympathize with the interviewers and express their true feelings and concerns. Such a situation occurred in the study of Leung et al., in which caregivers in the expressive writing group could speak with each other and share their difficulties [20]. Therefore, this might be a reason for the lack of significant effect of expressive writing in the current study that each caregiver did writing alone without any communication with researchers and other caregivers.

The main strength of our study was that it did show that family-based dignity intervention had a significant impact on anticipatory grief compared to the control group. Also, this was the first controlled clinical trial that examined the effects of both family-based dignity intervention and expressive writing on anticipatory grief in family caregivers of dying cancer patients. In addition to the effects of family-based dignity intervention and expressive writing alone, the combined effect of these interventions was assessed in a separate intervention group. Also, participants were allocated to the 4 intervention groups using block randomization. It seems that the sample size of this trial was adequate and provided sufficient power to detect the efficacy of interventions. Moreover, we controlled for baseline measurements in the statistical analysis. Some limitations of our study need to be taken into account. Despite using validated questionnaires for the assessment of anticipatory grief, misclassification of caregivers in terms of this variable cannot be fully excluded. Since the interventions used in the current study were interview-based, we cannot blind the caregivers to the interventions. Although we do block randomization, we could not match the intervention groups in terms of caregivers’ age and gender.

In conclusion, family-based dignity intervention presented by an experienced nurse improves anticipatory grief and its subscales in caregivers of dying cancer patients. However, we found no significant effect for expressive writing intervention and combined interventions of expressive writing and family-based dignity intervention. Since family-based dignity intervention can easily be carried out in palliative care centers, it can be taught to nurses to use in their conversations with cancer patients and caregivers. It must be kept in mind that family-based dignity intervention is not routinely conducted in cancer treatment centers in Iran.

## Data Availability

All data are available under a reasonable request.

## Abbreviations

RCT: Randomized controlled trial
AGS: Anticipatory grief scale
CV: Content validity (CV)
ITT: Intention-to-treat
ANOVA: Analysis of variance
